# Exploitation of response surface method for the optimization of RF-MEMS reconfigurable devices in view of future beyond-5G, 6G and super-IoT applications

**DOI:** 10.1038/s41598-022-07643-0

**Published:** 2022-03-03

**Authors:** Jacopo Iannacci, Girolamo Tagliapietra, Alessio Bucciarelli

**Affiliations:** 1 Center for Sensors and Devices (SD), Fondazione Bruno Kessler (FBK), 38123 Trento, Italy; 2 CNR-Nanotec, National Council or Research, Campus Ecotekne-Università del Salento, 73100 Lecce, Italy

**Keywords:** Nanoscale devices, Electrical and electronic engineering, Mechanical engineering, Computational science, Statistics

## Abstract

The emerging paradigms of the Beyond-5G, 6G and Super-IoT will demand for high-performance Radio Frequency (RF) passive components, and RF-MEMS technology, i.e. Microsystems-based RF passives, is a good candidate to meet such a challenge. As known, RF-MEMS have a complex behavior, that crosses different physical domains (mechanical; electrical; electromagnetic), making the whole design optimization and trimming phases particularly articulated and time consuming. In this work, we propose a novel design optimization approach based on the Response Surface Method (RSM) statistical methodology, focusing on a class of RF-MEMS-based programmable step power attenuators. The proposed method is validated both against physical simulations, performed with Finite Element Method (FEM) commercial software tools, as well as experimental measurements of physical devices. The case study here discussed features 3 DoFs (Degrees of Freedom), comprising both geometrical and material parameters, and aims to optimize the RF performances of the MEMS attenuator in terms of attenuation (S21 Scattering parameter) and reflection (VSWR—Voltage Standing Wave Ratio). When validated, the proposed RSM-based method allows avoiding physical FEM simulations, thus making the design optimization considerably faster and less complex, both in terms of time and computational load.

## Introduction

Significant part of current research in the fields of electronics, telecommunications and distributed sensing networks, falls under the umbrella of wide application paradigms, among which the Internet of Things (IoT)^1^, the Internet of Everything (IoE)^2^ and the 5G^3,4^ are undoubtedly dominating. Looking further ahead, in about one decade from now, the Super-IoT, equivalently addressed by the term Tactile Internet (IT), along with the 6G, will mark an unprecedented leap beyond common conceptions of applications and of Quality of Experience (QoE) made available to the end-user^5–7^. As it is straightforward to envision, 6G will demand for remarkable performances in terms of data transmission capacities. Taking as reference the currently under deployment 5G, the transition to the so-called Beyond-5G and then to the 6G, will mark a 1000 times increase of data rates, from the (already significant) 1 Gbps of the 5G, to 1 Tbps^6^. Apart from the huge requirements in terms of transmission/reception, other technical challenges will have to be addressed, like very-low End-To-End (E2E) latency, stepping from 5 ms for 5G, down to 1 ms for 6G, along with a very-high reliability of transmissions, these characteristics being crucial for safety critical applications, among which Vehicle-To-Vehicle (V2V) and Massive Machine-Type Communications (MMTC)^8^, as well as remote surgery^9^, are certainly valuable examples. From the technology point of view, the just mentioned specs will demand for venturing frequency ranges well-above 6 GHz, thereby including mm-waves (30–100 GHz), as well as the sub-THz range (100–300 GHz), necessary in turning Small/Tiny Cells^10^, massive-MIMO (Multiple-Input-Multiple-Output) and Large Intelligence Surface (LIS)^11^ antenna technologies into reality.

Given the scenario depicted up to now, current and future network and communication paradigms will massively capitalize on very-high performance, frequency agile and wideband Hardware (HW) components. To this end, the focus of the current contribution is on low-complexity Radio Frequency (RF) passive components, and in particular on MEMS (MicroElectroMechanical-Systems) technology for their realization, well-known in literature with the RF-MEMS acronym^12^. Across more than two decades of research, a broad variety of highly-miniaturized RF-MEMS-based passives with remarkable characteristics, in terms of RF performances and frequency wide-operability, has been demonstrated, like ohmic and capacitive micro-relays^13,14^, multi-state phase shifters^15^, tunable filters^16^, switching matrices^17,18^, and so on.

Differently from other consolidated technologies, MEMS always exhibit a complex multi-physical behavior, in which typical electrical and electronic properties of materials are coupled to the mechanical and mixed electromechanical domains. In particular, in the case here at stake of RF-MEMS, the structural/mechanical domain is coupled to electrostatics and electromagnetics^19^. This turns into an articulated and diverse set of Degrees of Freedom (DoFs) available to the designer, in order to optimize the electromechanical and RF characteristics of the studied RF-MEMS device, often revealing a non-negligible number of trade-offs across the mentioned physical domains. The approaches and techniques at hand to manage such complex optimization problems, are various and effective. Typically, a very good accuracy of the simulated results comes from commercial tools based on the Finite Element Method (FEM) analysis^20^, the ANSYS (www.ansys.com) and COMSOL (www.comsol.com) environments being the most commonly used. The main drawbacks of FEM are that the computational complexity of the model and the time of analysis can be considerable, especially if fine meshing is used to get higher accuracy and/or the geometry of the model is complex. Moreover, the available FEM tools are not suitable to simulate the whole multi-physical behavior of RF-MEMS. Therefore, it might be necessary using different environments, e.g. one for the electromechanical coupling, another for the RF properties, making the overall design optimization in the loop more tedious. In light of these considerations, there exist multi-domain simulation approaches based on simplified/compact analytical models, as well as on equivalent lumped element networks^21,22^, that enable fast simulation and DoFs assessment of RF-MEMS, at the cost of lower accuracy and reduced usability. As a matter of fact, the best practice is often that on using both tools, i.e. simplified models in the rough design evaluation phase, looking e.g. for the sensitivity of the available DoFs, and FEM tools for the fine optimization.

We chose as target device for this study an RF passive component that is quite critical for the MIMOs and 6G applications mentioned above, that is a multi-state RF power attenuator. A few design concepts, entirely realized in RF-MEMS technology, were already presented and discussed by some of the Authors, demonstrating good characteristics up to 110 GHz, and therefore providing a base of experimental data to be employed as reference for the novel predictive methodology discussed in the following pages.

Given such a frame, we propose an innovative design optimization approach, orthogonal with respect to the classical in use methodologies, that allowed us to predict the results of physical simulations without the need of performing them every time a parameter is varied. We based our approach on a Response Surface Method (RSM), that is a common statistical methodology, in which the system under observation is considered as a black box, with the controllable factors as inputs and the yields of interest as outputs. In the specific case here at stake, the inputs are related to geometry and materials parameters DoFs of the studied RF-MEMS design concept. On the other hand, since the device of interest is an RF power attenuator (as mentioned more in details below), the outputs of interests are the Scattering parameters (S-parameters), with particular focus on the transmission (S21), providing indications on the achieved level of attenuation, and the Voltage Standing Wave Ratio (VSWR), that is related to the amount of reflected power, i.e. dependent on the S11 parameter. RSM allows to build empirical equations that capture the behavior of the system within the considered range of the factorial space. As opposed to physical models, such equations can be applied regardless of the factors values, as long as the latter ones are interpolated within the observed ranges of data. Bearing this in mind, the great advantage of RSM is the general understanding of the yields trend, even in a wide range, by using few simulations performed in some strategical points.

In order to confirm the RSM method, we test it by simulating points inside the considered range but not used to build the empirical model, and, as further proof, against the values obtained by experimental measurements of a physical device. By building an RSM model on a small set of simulations, we prove its reliability in predicting with good accuracy the S21 and VSWR parameters, given the characteristics of the device geometry.

The paper is arranged as follows. The second section discusses the RF-MEMS step attenuator design concepts, reporting first on the technology and working principles, and then on the 3D FEM model of the multi-DoFs device (target of the subsequent RSM-based analysis), along with its validation against experimental datasets. The third section reports the development of the RSM optimization method on the basis of FEM datasets as inputs, and its validation and confirmation with respect to additional FEM simulations and experimental data. In last section, eventually, collects some conclusive considerations.

## RF-MEMS reconfigurable attenuator modules

**Figure 1:**
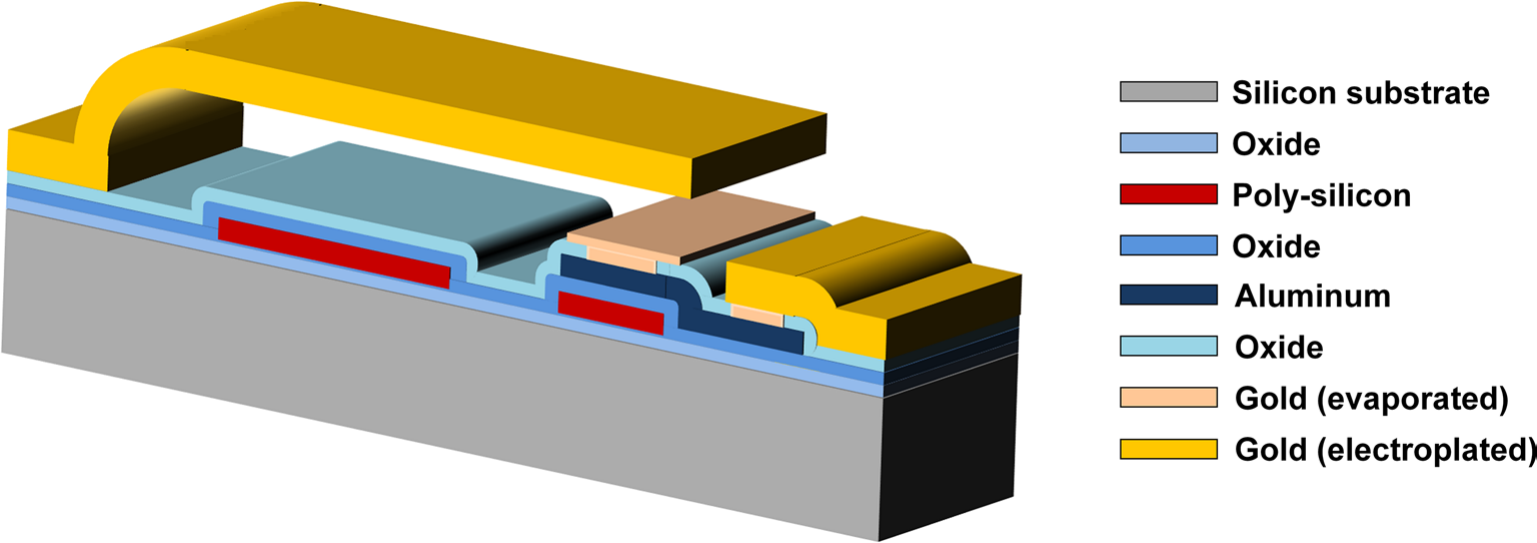
Cross-section of the RF-MEMS technology employed in this study. Image created with Microsoft Office 365 PowerPoint (www.office.com).

The devices discussed in this work are realized in an RF-MEMS technology platform based on a surface micromachining process, whose details are discussed in^23^. A cross-sectional view of the process is shown in Fig. 1, and it employs two conductive thin-film layers protected by oxide, i.e. polycrystalline silicon (poly-silicon) and aluminum, above which the actual MEMS suspended electrostatically-driven membranes are realized in electroplated gold. Moreover, a thin-film of evaporated gold is exploited to reduce the metal-to-metal contact resistance.

Starting from the mentioned technology, the RF-MEMS attenuators design concepts at stake in this work are going to be introduced. In particular, the following “Design concepts, micro-fabrication and characterization of 1-bit building blocks” reports the working principles, experimental characterization and the validation of FEM simulations, with reference to two 1-bit series and shunt dual devices. Given this set of data, a multi-parametric 2-bit RF-MEMS attenuator concept is then discussed in “2-bit composed attenuator module concept and parametric analysis”. The latter device will be the case study of the RSM analysis, subsequently reported in “Response surface method (RSM)”.

### Design concepts, micro-fabrication and characterization of 1-bit building blocks

**Figure 2:**
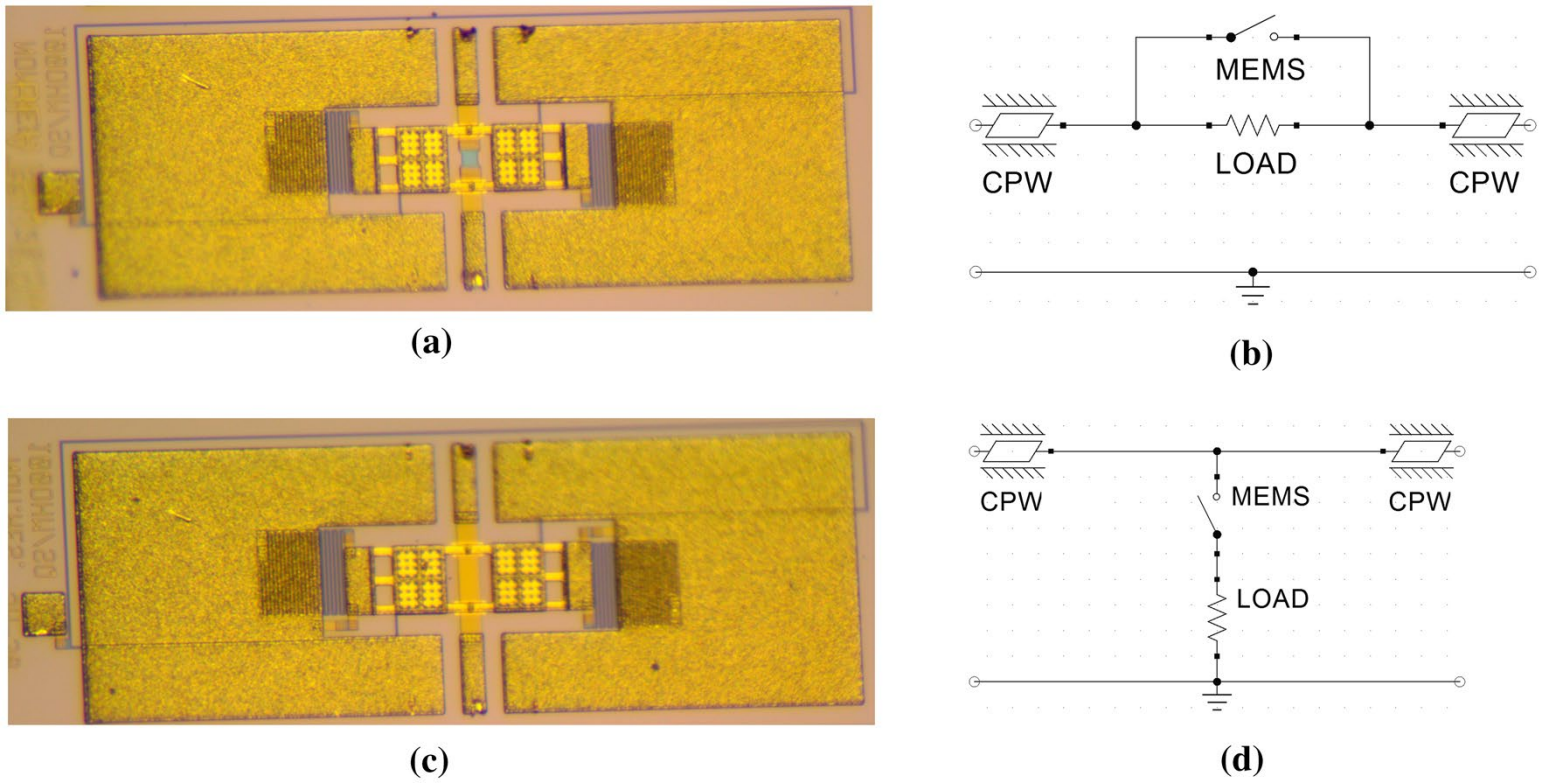
Microphotograph of the series (**a**) and shunt (**c**) RF-MEMS 1-bit attenuator samples, and corresponding equivalent lumped element circuits of the series (**b**) and shunt (**d**) design concepts^24^. Images (**b**) and (**d**) created with Quite Universal Circuit Simulator (QUCS) 0.0.19 (http://qucs.sourceforge.net).

The starting point of this study is a set of two 1-bit attenuator design concepts, realized in the RF-MEMS technology mentioned above. Both devices are framed within a Coplanar Waveguide (CPW) configuration of 2 mm by 1.7 mm, and feature an electrostatically controlled clamped–clamped series ohmic switch for introducing or avoiding attenuation of the RF signal. In both cases, the attenuation is caused by a resistive load, realized with the 630 nm-thick poly-silicon buried layer (see Fig. 1). The microphotographs of both the design concepts, along with the corresponding equivalent lumped element circuits, are reported in Fig. 2 and discussed in^24^.

Figure 2a shows the microphotograph of the series 1-bit attenuator. The poly-silicon resistive load is inserted in series on the RF line. Therefore, when the MEMS switch is not actuated (OFF state), the resistor attenuates the RF signal. Differently, when the MEMS micro-relay is pulled-in (ON state), the resistive load is shorted by the MEMS membrane, as visible in the equivalent network in Fig. 2b. The dual design concept is the shunt 1-bit attenuator, shown in Fig. 2c. In this case, a low-resistivity underpass connects the RF input and output. The resistive load consists of two poly-silicon parallel resistors, connecting the MEMS switch membrane to both the RF ground planes. This means that when the MEMS micro-relay is OFF, the RF signal flows unattenuated through the device. On the other hand, when the MEMS switch is ON, the resistive loads to RF ground are inserted, thus attenuating the signal, as visible in the equivalent network in Fig. 2d. The poly-silicon layer used for the resistive loads has a resistivity (R_SQ_) of 140 Ω/sq. Having said that, the series 1-bit design features a resistor with length and width of 45 µm and 40 µm, respectively, therefore yielding a load of 170 Ω. The shunt attenuator, instead, features two resistors in parallel with length and width of 15 µm and 25 µm (42 Ω, each), respectively, leading to a load of 21 Ω.

**Figure 3:**
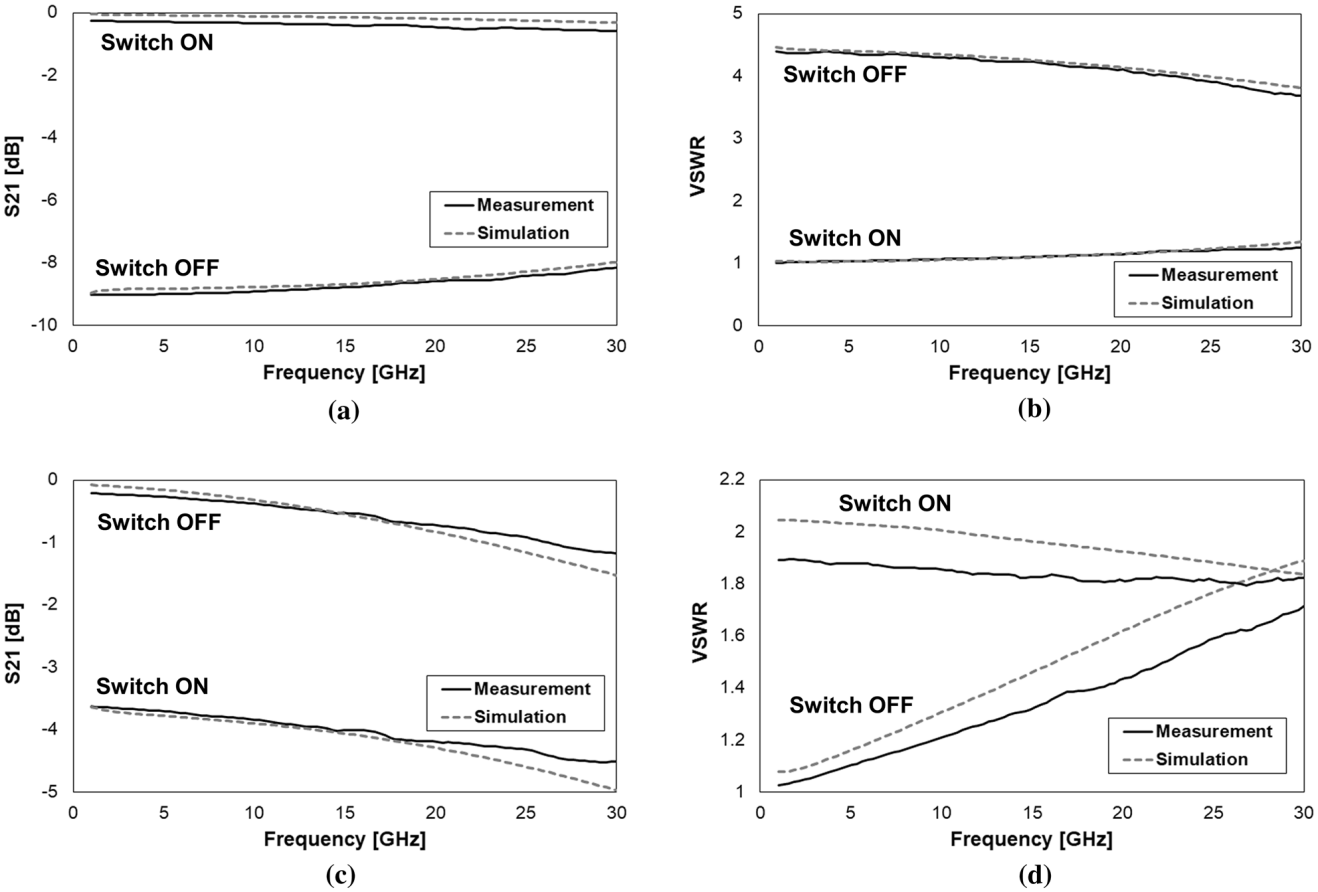
Comparison of the measured and simulated S-parameters characteristics of the 1-bit series (**a**,**b**) and shunt (**c**,**d**) RF-MEMS attenuators from 1 to 30 GHz. S21 (a) and VSWR (b) of the series attenuator, when the resistive load is inserted (switch OFF) and shorted (switch ON). S21 (c) and VSWR (d) of the shunt attenuator, when the resistive load is inserted (switch ON) and not inserted (switch OFF). Images created with Microsoft Office 365 Excel (www.office.com).

Despite both the mentioned design concepts were experimentally tested up to 110 GHz^24^, for the purposes of this work we limit the frequency range of interest between 1 and 30 GHz, as the attenuation levels exhibit a particularly flat (nearly-linear) characteristic. In light of these considerations, full-3D models of the 1-bit devices in Fig. 2a, c are built within the Ansys HFSS Finite Element Method (FEM) RF simulation environment for validation purposes^25^. The comparison of the measured and simulated S-parameters (Scattering parameters) characteristics of the transmission/attenuation (S21 parameter) and Voltage Standing Wave Ratio (VSWR), for both the series and shunt 1-bit devices in the ON and OFF micro-relay configurations, are reported in Fig. 3.

Looking at the plots in Fig. 3 it is possible to notice the pronounced accuracy of the FEM models in predicting the behavior of both the S21 and VSWR, in the ON/OFF configurations of the series and shunt 1-bit design variations. Moreover, is must also be stressed that all the observed traces are rather flat, with a nearly-linear behavior, across the whole observed frequency range. In fact, the plots in Fig. 3c,d (especially the latter one), might suggest a less accurate match between simulations and measurements, if compared to Fig. 3a,b. However, it must be stressed that the difference, in terms of vertical axes range, between the ON and OFF state of the shunt attenuator is smaller than in the case of the series device. In particular, such a range is of 10 dB and 5 for the S21 and VSWR of the series device, respectively, while it is of 6 dB and 1.1 for the shunt one. This makes the differences of the measured and simulated traces look more compressed in the case of the series device, and more enhanced, on the other side, for the shunt attenuator. What has to be stressed is that the displacement of the simulated traces is always ranging between a few tenths of dB and 1 dB at most, with a constant good match with the qualitative behavior of the experimental traces.

### 2-bit composed attenuator module concept and parametric analysis

**Figure 4:**
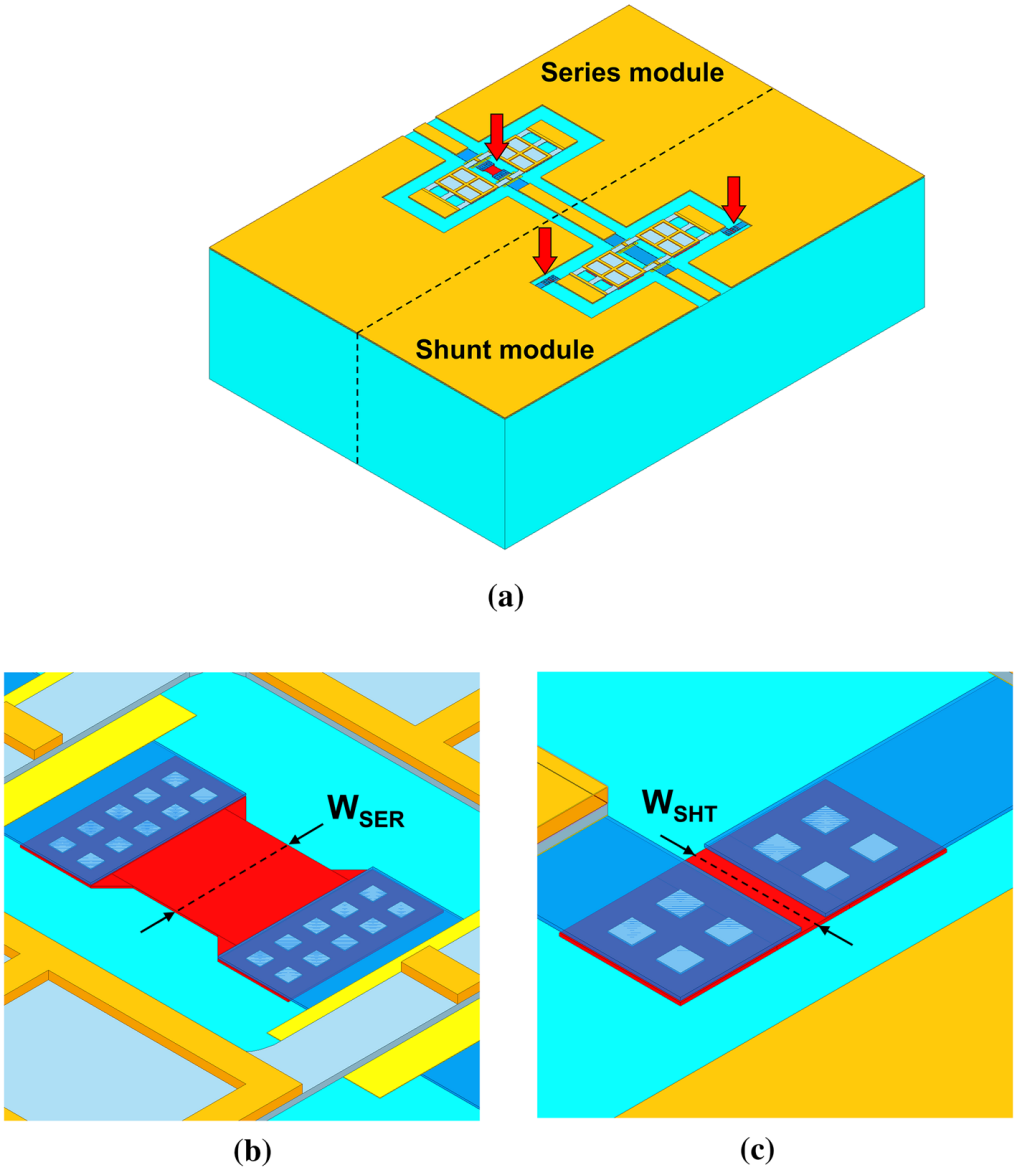
Ansys HFSS full-3D model of the 2-bit RF-MEMS attenuator (**a**). Close-up of the poly-silicon resistor/s in the series (**b**) and shunt (**c**) section of the composed attenuator. Images created with Ansys Electronics Desktop 2020 R2, HFSS (www.ansys.com/products/electronics).

Starting from the validated FEM modelling approach previously discussed, a 2-bit composed attenuator concept is here introduced, and it will be the basis for the RSM-based analysis developed in the next section. The 2-bit device features the series and shunt 1-bit RF-MEMS modules (reported above) connected to each other, forming a unique network. A full-3D model is built for the whole device (see Fig. 4a), in which the widths of the poly-silicon resistors are parameterized. The close-ups in Fig. 4b,c highlight where the poly-silicon resistors are located, with focus on the width of the series and shunt sections loads, respectively labeled as W_SER_ and W_SHT_.

**Figure 5:**
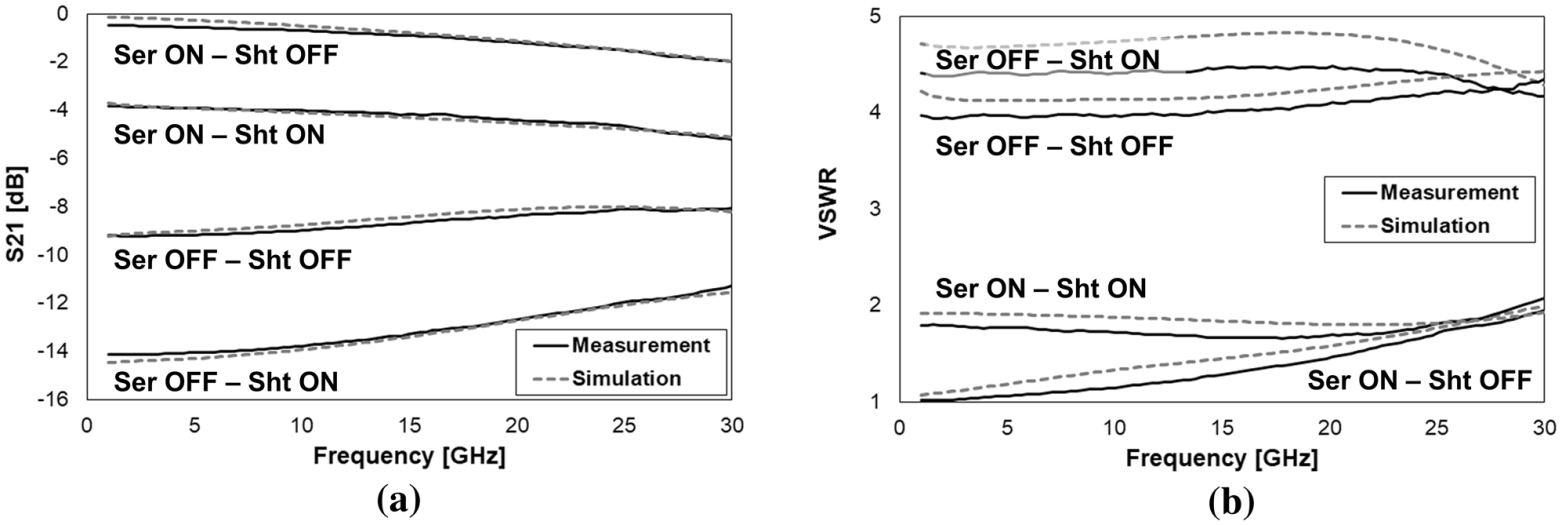
Comparison of the measured and simulated S21 (**a**) and VSWR (**b**) of the 2-bit RF-MEMS attenuator in all the four implemented network configurations. Images created with Microsoft Office 365 Excel (www.office.com).

The model is further validated against experiments. To do so, the S-parameters measured datasets of the series and shunt modules with the MEMS switches ON and OFF, are cascaded to each other, realizing all the four possible combinations. The full-3D model is then simulated in the same four configurations, while keeping the poly-silicon resistors as described before. The results are summarized in Fig. 5.

As visible in both plots, the FEM model predicts rather accurately the S21 and VSWR characteristics of the 2-bit network in all the four configurations. Concerning the apparent lower accuracy of simulations in the plot in Fig. 5b, the same considerations previously developed when discussing Fig. 3, hold validity in this case, as well. Starting from the just validated model, the widths of the resistors in the series and shunt subsections (W_SER_ and W_SHT_) and the resistivity of the poly-silicon layer (R_SQ_) are parameterized and modified, in order to carry on the 3 DoFs RSM analysis discussed in the following “Response surface method (RSM)”.

## Response surface method (RSM)

In order to interpolate an empirical model, a Response Surface Method (RSM), previously exploited by some of the Authors in solving different problems^26–28^, has been adopted. The entire statistical analysis was performed by using the programming language R^29^, and following the statistical strategy previously described in^30,31^. An initial comparison to verify the presence of significant differences among the various analyzed groups, based on the Analysis of Variance (ANOVA), then followed by a Turkey multi-comparison test^26,28,31^, were performed. The levels of significance were assigned as follows: *p* ≤ *0.1* (.), *p* ≤ *0.05* (*), *p* ≤ *0.01* (**), *p* ≤ *0.001* (***). We considered three continuous factors, two geometrical, W_SER_ in mm (Factor B) and W_SHT_ in mm (Factor C), and one physical, the resistance R_SQ_ in Ω/sq (Factor A). For each factor, three levels were selected and all the possible combinations among them are simulated. The outcomes of the simulation are the S21 (in dB) and the VSWR (dimensionless) curves versus frequency in the 1–30 GHz range. These curves were then fitted by a linear function, the slope and intercept extracted and used as yields in the RSM analysis. It should be noted that RSM might be used also to reconstruct the entire curve by adding the frequency as a factor. To this end, Machine Learning (ML) may be applied to reproduce the entire simulated curves, however without the advantage of obtaining an empirical equation. The dataset extracted from the simulations used to perform the statistical analysis, is reported in Table 1.

**Table 1:** Dataset of the simulations results used to build the empirical model based on RSM.

R_SQ_ [Ω/sq]	W_SER_ [mm]	W_SHT_ [mm]	S21 intercept [dB]	S21 slope [dB/GHz]	VSWR intercept [dB]	VSWR slope [dB/GHz]
40	0.03	0.015	− 12.67216652	0.059350727	1.188205649	0.097160264
40	0.03	0.025	− 15.39458966	0.089605739	0.972075466	0.124513722
40	0.03	0.035	− 16.08886316	0.098023383	0.945825946	0.127134706
40	0.04	0.015	− 11.92027676	0.043096893	0.995124841	0.105262461
40	0.04	0.025	− 14.57882223	0.072414756	0.796383088	0.131821102
40	0.04	0.035	− 14.79492263	0.066532712	0.671733869	0.144031128
40	0.05	0.015	− 11.37529328	0.03153613	0.913797137	0.109097486
40	0.05	0.025	− 13.82488701	0.052610155	0.68049833	0.138392045
40	0.05	0.035	− 14.19458044	0.056826687	0.654965362	0.143010608
100	0.03	0.015	− 13.54311748	0.082555714	3.684737412	0.013429519
100	0.03	0.025	− 15.7317411	0.117521792	3.561574471	0.025688654
100	0.03	0.035	− 16.40119334	0.126477712	3.634209791	0.027418366
100	0.04	0.015	− 12.60087694	0.072809245	3.200414769	0.023016386
100	0.04	0.025	− 14.64398539	0.103617343	2.970855054	0.036708921
100	0.04	0.035	− 15.08853544	0.108208837	2.92277708	0.04100924
100	0.05	0.015	− 11.60803964	0.060849099	2.712278938	0.030961366
100	0.05	0.025	− 13.55013972	0.086344864	2.478462182	0.046268654
100	0.05	0.035	− 14.07875106	0.09320415	2.482899185	0.048654383
160	0.03	0.015	− 15.12992736	0.0880637	6.056665162	− 0.030828118
160	0.03	0.025	− 16.79049269	0.11946873	5.913736916	− 0.022748117
160	0.03	0.035	− 17.57502719	0.133268161	6.009391688	− 0.021958681
160	0.04	0.015	− 13.7599272	0.070563827	5.061887249	− 0.017457183
160	0.04	0.025	− 15.6869909	0.111180749	4.959185097	− 0.005752735
160	0.04	0.035	− 16.16036029	0.114033122	4.949123279	− 0.005214859
160	0.05	0.015	− 12.80918354	0.063837424	4.388835413	− 0.005650973
160	0.05	0.025	− 14.4574395	0.095453089	4.203035839	0.006482902
160	0.05	0.035	− 15.10005063	0.10384427	4.213072729	0.007184443

Starting from the simulated curves, their intercept and slope were calculated in the 1–30 GHz range and used as yields. The reference configuration is that with both the resistive loads of the RF-MEMS network in Fig. 4 inserted, that is when the micro-switches of the series and shunt sections are OFF and ON, respectively.

Moreover, the complete model that can be obtained considering the three levels for each factor is reported in the following Eq. (1).

1 $$ \begin{aligned}F\left(Y\right)&={c}_{0}+{c}_{1}A+{c}_{2}B+{c}_{3}C+{c}_{4}AB+{c}_{5}AC+{c}_{6}BC+{c}_{7}ABC+{c}_{8}{A}^{2}+{c}_{9}{B}^{2}+{c}_{10}{C}^{2}\\&\quad+{c}_{11}{A}^{2}B+{c}_{12}{A}^{2}C+{c}_{13}A{B}^{2}+{c}_{14}A{C}^{2}+{c}_{15}B{C}^{2}+{c}_{16}{B}^{2}C+{c}_{17}AB{C}^{2}\\&\quad+{c}_{18}A{B}^{2}C+{c}_{19}{A}^{2}BC+{c}_{20}{A}^{2}{B}^{2}C+{c}_{21}{A}^{2}B{C}^{2}+{c}_{22}A{B}^{2}{C}^{2}+{c}_{23}{A}^{2}{B}^{2}{C}^{2}\end{aligned} $$

An ANOVA test followed by a Turkey multi-comparison was conducted to verify the significance of each term in the reported equation. Only the terms with a significant effect (*p* ≤ 0.01) were included in the model. The function F has been chosen to both normalize the model residues and to make them pattern-less. The model was considered significant with a *p*-value ≤ 0.05. To determine the model goodness of fit, the coefficient of determination (*R*^*2*^) was calculated. Models with a perfect fitting are characterized by *R*^*2*^ = 1.

### RSM empirical models

**Figure 6:**
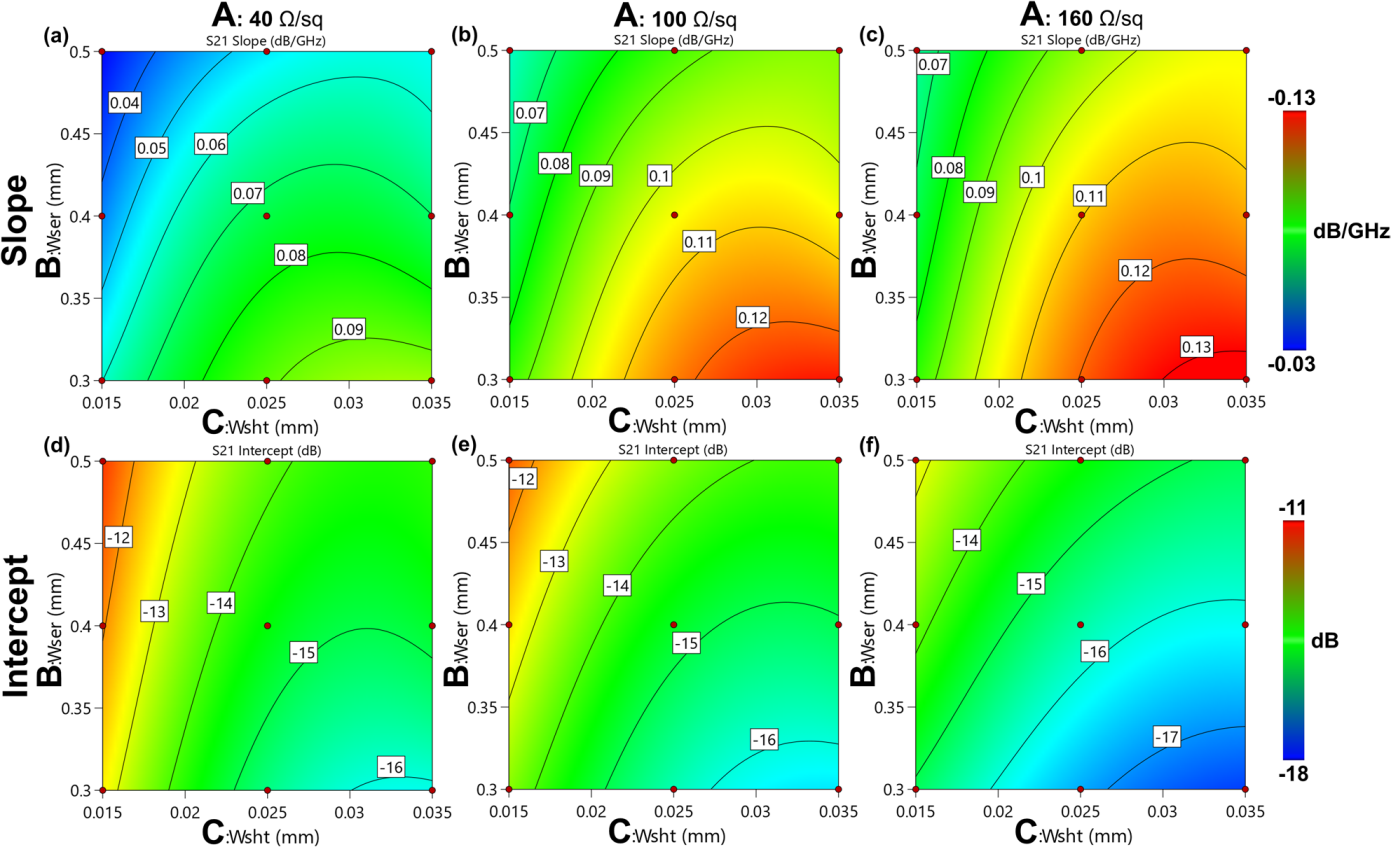
Contour plots (**a**–**c**) of the empirical model for the slope and the intercept (**d**–**f**) of the S21 curves in the linear zone, obtained from the physical simulations. The red points indicate where the physical simulations are performed. Image created with R v.4.0.4 using as Integrated Development Environment (IDE) R Studio v. 1.4.1104 (www.rstudio.com/products/rstudio) and GGPlot2 3.3.5 as graphic package (https://ggplot2.tidyverse.org). The graphs were then mounted in their final version using Affinity Designer v. 1.10 (https://affinity.serif.com/en-us).

The RSM models of the slope (Eq. 2) and intercept (Eq. 3) of the S21 curves are shown in the first and second row of Fig. 6, respectively, as contour plots. Since the models are four-dimensional, the contour plots are sliced with respect to the three resistance levels used for the simulations. As it can be inferred by the plots, neither the slope, nor the intercept are linear inside the considered range. The curvature is the result of the significance of both the several non-linear mixed and squared terms, as clearly visible in the ANOVA Tables S2 and S3, available in the supplementary material provided with this paper.

2 $$ \begin{aligned}{m}_{S21}&=0.457261+0.000725081A - 2.66008B -35.1573C + 0.000393626AB + 0.00573355AC + 228.089BC \\ &\quad  - 3.60289{*10}^{-6}{A}^{2}+ 3.20619{B}^{2} + 801.457{C}^{2} - 291.759{B}^{2}C - 4889.91B{C}^{2} + 6182.54{B}^{2}{C}^{2}\end{aligned} $$

3 $$ \begin{aligned}{q}_{S21}&=-16.3748-0.025158A+69.3352B+187.384C-0.117305AB+4.92259AC \\ &\quad-5298.16BC-0.00039027{A}^{2}-85.637{B}^{2}-14189.1{C}^{2}-10.1165ABC \\ &\quad + 0.00184065{A}^{2}B-0.00142162{A}^{2}C+0.273805A{B}^{2}-44.6545A{C}^{2} \\ &\quad + 6817.34{B}^{2}C+138431B{C}^{2}-0.00258687{A}^{2}{B}^{2}+11.4915A{B}^{2}C -172251{B}^{2}{C}^{2}\end{aligned} $$

**Figure 7:**
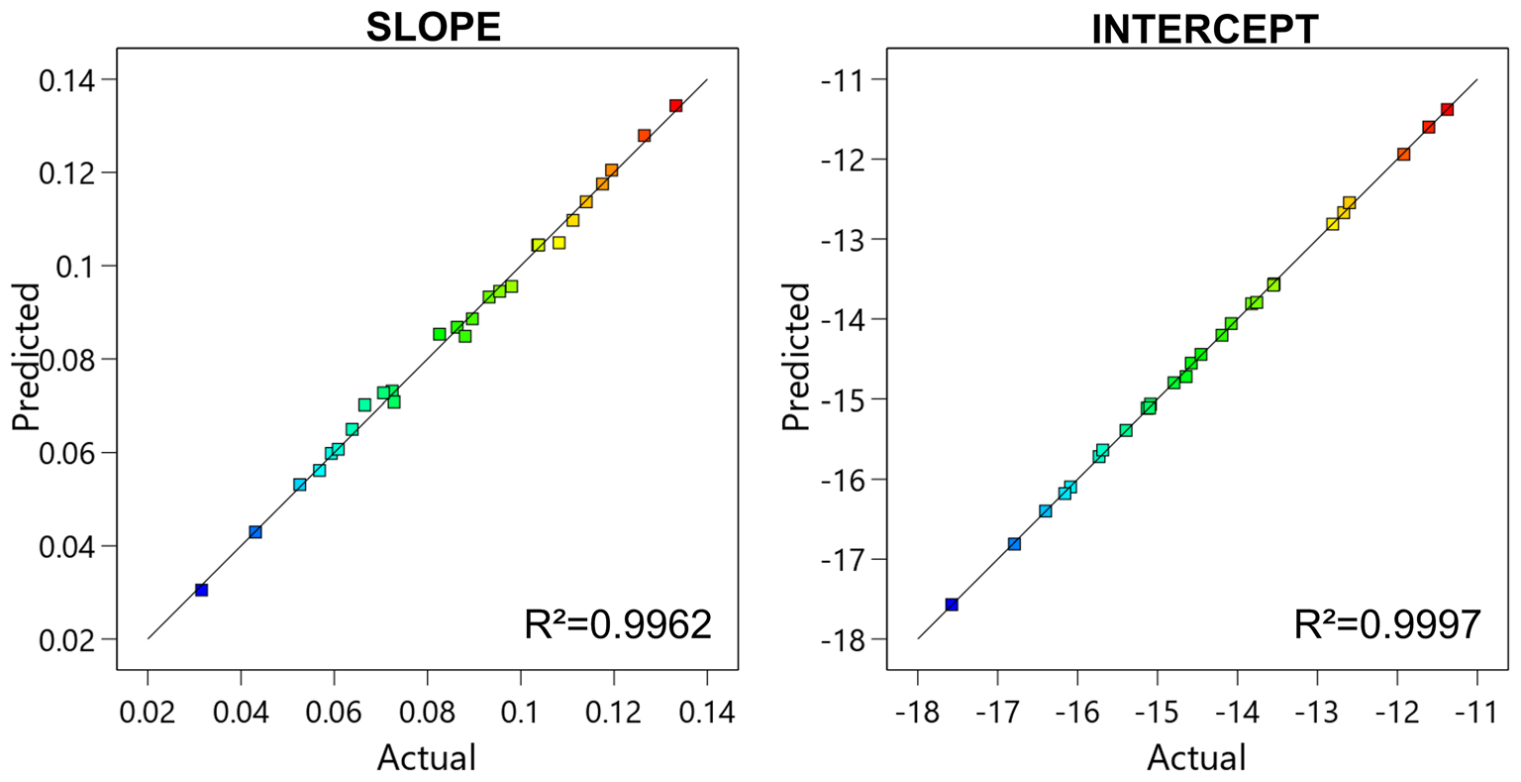
Actual versus predicted plot of the S21 slope and intercept. In both cases, the points are slightly scattered on the diagonal line, indicating a good agreement between the RSM and the physical models. This was also confirmed by the value of *R*^*2*^ close to 1. Image created with R v.4.0.4 using as Integrated Development Environment (IDE) R Studio v. 1.4.1104 (www.rstudio.com/products/rstudio) and GGPlot2 3.3.5 as graphic package (https://ggplot2.tidyverse.org). The graphs were then mounted in their final version using Affinity Designer v. 1.10 (https://affinity.serif.com/en-us).

Both the proposed models almost perfectly fitted the values obtained by simulation and linearization, as it is evident by observing Fig. 7, in which the values obtained by physical simulations are reported against the values obtained using the RSM predictive model. In both the slope and intercept graphs, the points follow the diagonal line, indicating a good agreement of the empirical and physical models. This was also confirmed by the value of *R*^*2*^ that was close to 1, thus indicating a direct relationship between the physical and the RSM model.

**Figure 8:**
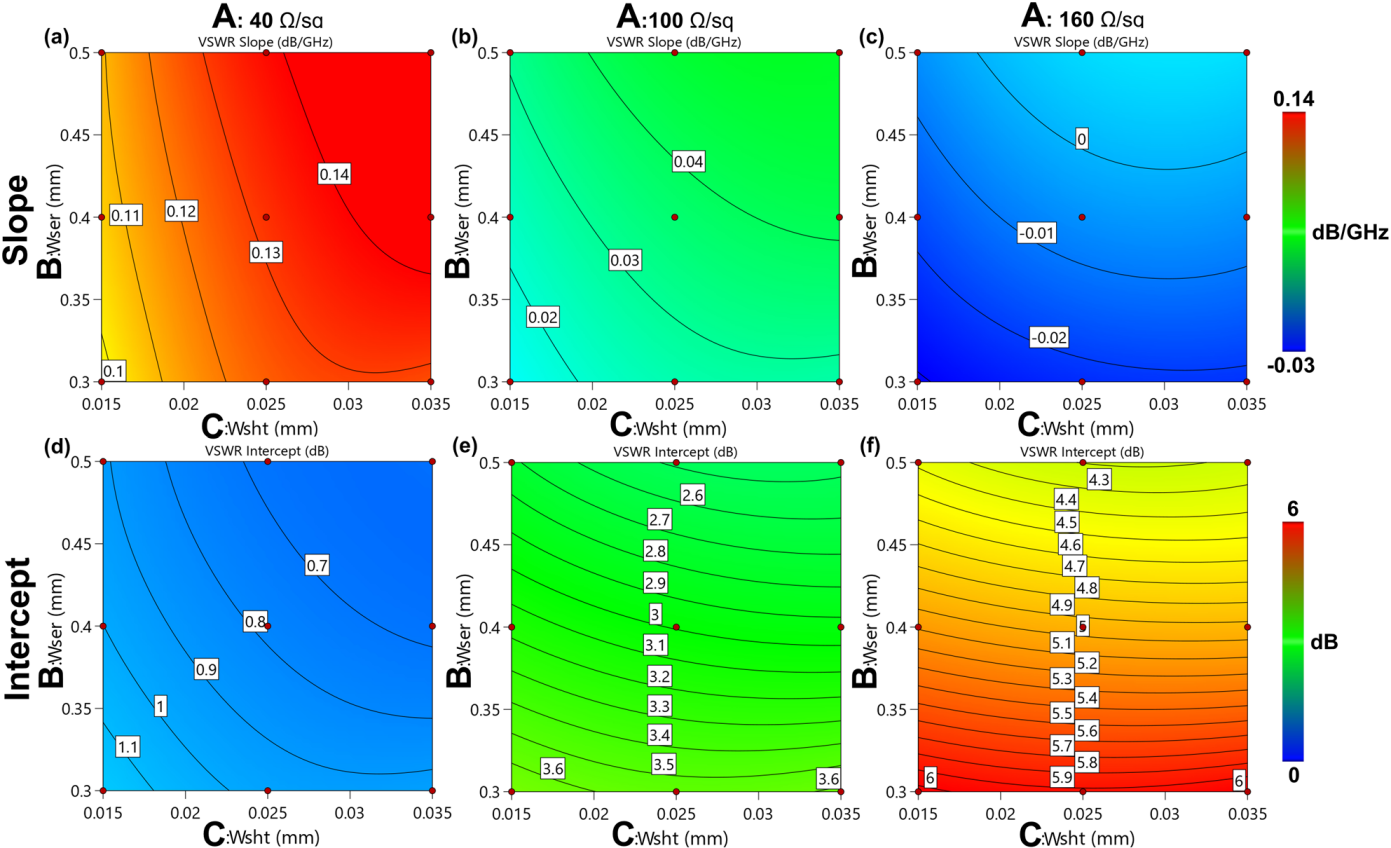
Contour plots (**a**–**c**) of the empirical model for the slope and the intercept (**d**–**f**) of the VSWR curves in the linear zone, obtained from the physical simulations. The red points indicate where the physical simulations are performed. Image created with R v.4.0.4 using as Integrated Development Environment (IDE) R Studio v. 1.4.1104 (www.rstudio.com/products/rstudio) and GGPlot2 3.3.5 as graphic package (https://ggplot2.tidyverse.org). The graphs were then mounted in their final version using Affinity Designer v. 1.10 (https://affinity.serif.com/en-us).

The same procedure was adopted for the VSWR curves of the slope (Eq. 4) and intercept (Eq. 5). The models are presented in Fig. 8 as contour plots. Also in this case, the non-linearity was the result of the significant mixed and squared terms in the ANOVA (Table S4 for the slope and Table S5 for the intercept, in the supplementary material) and thus reported into the models equations.

4 $$ \begin{aligned}{\mathrm{m}}_{\mathrm{VSWR}}&=-0.506741+0.00409162\mathrm{A}+2.86928\mathrm{B}+57.8868\mathrm{C}-0.0254143\mathrm{AB}\\ &\quad-0.486292\mathrm{AC }-244.011\mathrm{BC}-5.11306*{10}^{-6}{\mathrm{A}}^{2}-3.52337{\mathrm{B}}^{2}-1234.97{\mathrm{C}}^{2}\\ &\quad +1.88941\mathrm{ABC}+3.02067*{10}^{-5}{\mathrm{A}}^{2}\mathrm{B}+0.000409849{\mathrm{A}}^{2}\mathrm{C}+0.0315036{\mathrm{AB}}^{2}\\ &\quad+9.88405{\mathrm{AC}}^{2}+304.123 {\mathrm{B}}^{2}\mathrm{C}+5538.24{\mathrm{BC}}^{2}-3.5864*{10}^{-5}{\mathrm{A}}^{2}{\mathrm{B}}^{2}\\ &\quad-0.00647226{\mathrm{A}}^{2}{\mathrm{C}}^{2}-2.30703{\mathrm{AB}}^{2}\mathrm{C}-41.4104\mathrm{ AB}{\mathrm{C}}^{2}-6880.94{\mathrm{B}}^{2}{\mathrm{C}}^{2}\\ &\quad+50.6698{\mathrm{AB}}^{2}{\mathrm{C}}^{2}\end{aligned} $$

5 $${q}_{VSWR}=4.78304+0.0226137A-24.1267B-389.905C+0.119356AB+0.77324AC +1661.85BC+0.000206589{A}^{2}+30.5411{B}^{2}+8117.56{C}^{2}-1.71093ABC-0.00109828{A}^{2}B-0.00305993{A}^{2}C-0.192804A{B}^{2}-2020.84{B}^{2}C-37832.6B{C}^{2}+0.00125253{A}^{2}{B}^{2}+0.0073876{A}^{2}BC+47474.1{B}^{2}{C}^{2}$$

**Figure 9:**
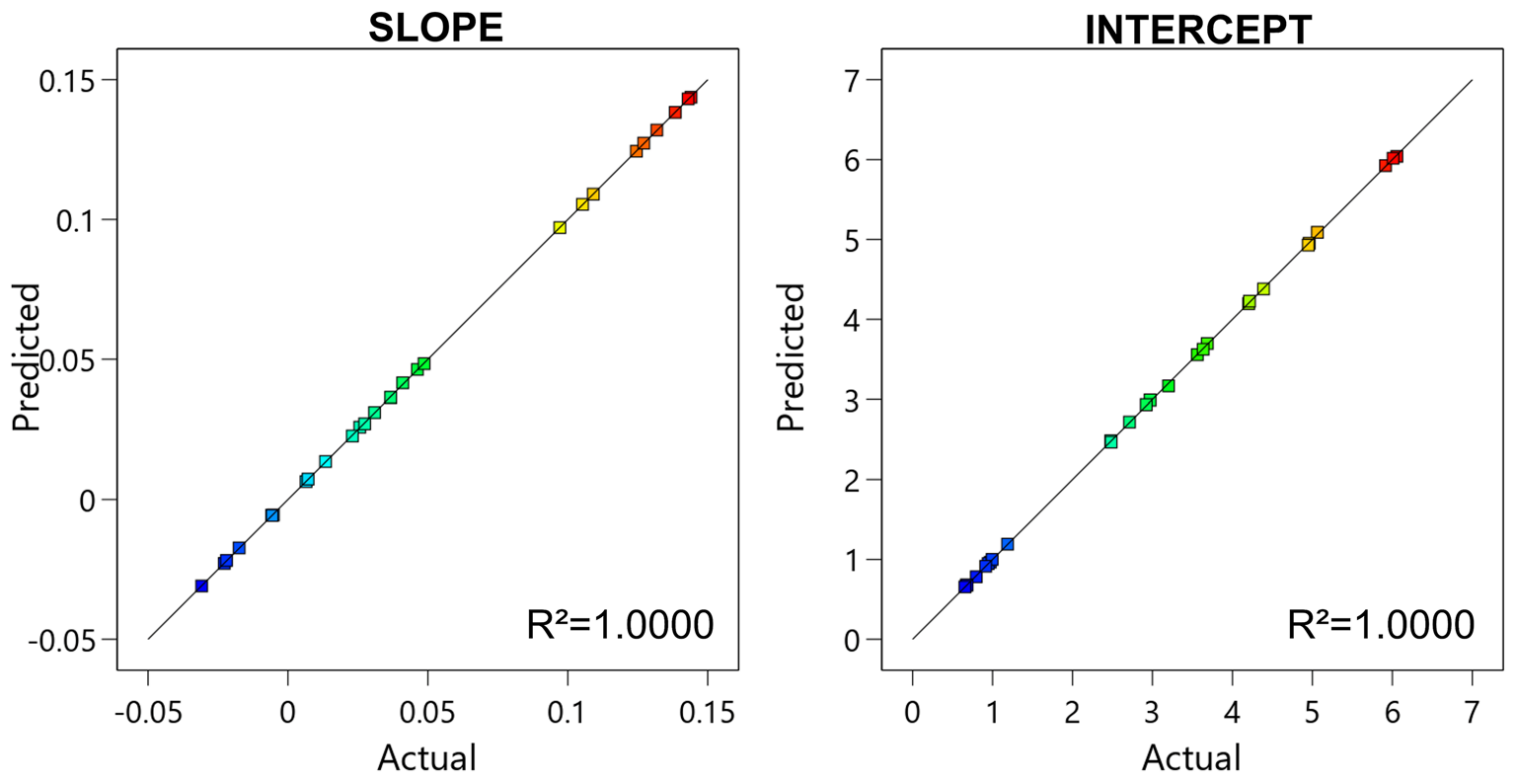
Actual versus predicted plot of the VSWR slope and intercept. In both cases, the points are perfectly on the diagonal line, indicating perfect agreement between the RSM and the physical models. This was also confirmed by the value of *R*^*2*^ equal to 1. Image created with R v.4.0.4 using as Integrated Development Environment (IDE) R Studio v. 1.4.1104 (www.rstudio.com/products/rstudio) and GGPlot2 3.3.5 as graphic package (https://ggplot2.tidyverse.org). The graphs were then mounted in their final version using Affinity Designer v. 1.10 (https://affinity.serif.com/en-us).

Figure 9 confirmed the very good agreement between the values extrapolated by the physical and the RSM models. Such a satisfactory correspondence rarely occurs if the data points come from real devices, and it is a condition that typically makes the model suspicious. However, in this case the data points were obtained from simulations and it is possible that the empirical model was able to effectively catch the underling physical model.

### Model confirmation

As confirmation points of the proposed RSM models, we simulated an additional set of eight points inside the considered factor range. The slope and the intercept extrapolated from the physical simulations were compared with the outcome obtained by the empirical RSM equations. The results are presented in Table 2 for the S21 parameter and in Table 3 for the VSWR.

**Table 2:** Results of the physically simulated and RSM data with respect to the confirmation points for the S21 parameter.

Factors			Phys. Sim	RSM	m Diff. [%]	Phys. Sim	RSM	q Diff. [%]
R_SQ_ [Ω/sq]	W_SER_ [mm]	W_SHT_ [mm]	m S21 [dB/GHz]	m S21 [dB/GHz]		q S21 [dB]	q S21 [dB]	
70	0.035	0.02	0.086	0.087	0.142	− 13.673	− 14.050	2.759
70	0.035	0.03	0.107	0.104	− 2.489	− 15.248	− 15.468	1.445
70	0.045	0.02	0.072	0.072	− 0.736	− 12.797	− 13.160	2.843
70	0.045	0.03	0.087	0.087	0.308	− 14.024	− 14.456	3.078
130	0.035	0.02	0.101	0.102	1.397	− 14.807	− 14.831	0.163
130	0.035	0.03	0.119	0.123	3.451	− 16.033	− 16.086	0.330
130	0.045	0.02	0.084	0.089	6.100	− 13.547	− 13.716	1.249
130	0.045	0.03	0.104	0.108	3.751	− 15.095	− 14.904	− 1.271

**Table 3:** Results of the physically simulated and RSM data with respect to the confirmation points for the VSWR parameter.

Factors			Phys. Sim	RSM	m Diff. [%]	Phys. Sim	RSM	q Diff. [%]
R_SQ_ [Ω/sq]	W_SER_ [mm]	W_SHT_ [mm]	m VSWR [dB/GHz]	m VSWR [dB/GHz]		q VSWR [dB]	q VSWR [dB]	
70	0.035	0.02	0.060	0.066	8.698	2.100	2.137	1.775
70	0.035	0.03	0.069	0.079	13.399	2.065	2.035	− 1.479
70	0.045	0.02	0.069	0.074	7.958	1.793	1.817	1.342
70	0.045	0.03	0.079	0.087	10.620	1.721	1.688	− 1.919
130	0.035	0.02	0.004	− 0.001	− 125.212	4.346	4.404	1.333
130	0.035	0.03	0.009	0.007	− 26.275	4.301	4.342	0.953
130	0.045	0.02	0.015	0.011	− 24.962	3.632	3.724	2.552
130	0.045	0.03	0.020	0.019	− 5.986	3.699	3.636	− 1.714

For the S21 angular coefficient parameters, the difference between the RSM and the physical model did not exceed the 7% for the slope and the 4% for the intercept. For the VSWR slope, the difference reached the 125% while for the intercept it did not exceed the 3%. In several cases the angular coefficient was close to zero, thus even small differences gave high differences when expressed in terms of percentage. The model still should be considered as valid.

In summary, the RSM model was validated against the full-3D FEM results generated by the model in Fig. 4, after having verified the latter against a set of experimental data, as reported in Fig. 5. In particular, for what concerns the R_SQ_ DoF, we performed the RSM validation against a few values, ranging from 40 Ω/sq and 160 Ω/sq, without including the R_SQ_ of the experimental data in Fig. 5, that is 140 Ω/sq. As further validation step, we decided to test the RSM model also against the R_SQ_ of the experiments, keeping of course W_SER_ and W_SHT_ to the nominal values, as they belong to the RF-MEMS physical sample. The results are shown in Table 4.

**Table 4:** Comparison between an experimental measurement conducted on the device, the extrapolation from simulation and the RSM model.

	Factors			Measured	Simulated	RSM	Measured	Simulated	RSM
	R_SQ_ [Ω/sq]	W_SER_ [mm]	W_SHT_ [mm]	m S21 [dB/GHz]	m S21 [dB/GHz]	m S21 [dB/GHz]	q S21 [dB]	q S21 [dB]	q S21 [dB]
	140	0.04	0.025	0.103	0.109	0.109	− 14.641	− 14.878	− 15.145
% Diff_MEAS_					6.238	6.559		1.615	3.444
% Diff_SIMU_						0.303			1.800

In the last two lines, the percentage difference with the measured and simulated data is reported.

The RSM was effective in predicting both the S21 slope and intercept of the measured device within a percentage of 3.5%. The simulated values were closer, within a percentage difference of 1.6%. However the proposed method allowed us to predict the value without running a simulation but simply by substituting the factor values into the model equations.

## Conclusion

In this work, we proposed an innovative design optimization approach, orthogonal with respect to classical methodologies, based on the Response Surface Method (RSM), i.e. a common statistical methodology in which the system under observation is considered as a black box, with the controllable factors as inputs and the yields of interest as outputs.

We focused our study bearing in mind the emerging/future paradigms of 5G, Beyond-5G, 6G and Super-Internet of Things (Super-IoT), approaching such a wide scenario from the low-complexity Hardware (HW) components point of view. In particular, we chose RF-MEMS technology as case study, i.e. MicroElectroMechanical-Systems (MEMS) for Radio Frequency (RF) passive components, given their multi-physical behavior (mechanical/structural, electrostatic, electromechanical and electromagnetic), along with the plethora of cross-domain Degrees of Freedom (DoFs) available for optimization.

The device at stake in this work is a multi-state RF power attenuator based on MEMS technology. We first built a full-3D Finite Element Method (FEM) model of the attenuator for the simulation of the attenuation (S21 parameter) and Voltage Standing Wave Ratio (VSWR), and then we validated the model against measured S-parameters (Scattering parameters) datasets. Subsequently, we chose three continuous factors influencing the levels of attenuation implemented by the device, i.e. two geometrical dimensions and the resistance of the thin-film layer realizing the load resistors. For each factor, we selected three values and we performed FEM simulations in all the 27 possible configurations. Given the pronounced flatness of the S21 response, we limited the study in the 1–30 GHz frequency range. Such curves were then fitted by a linear function, and the extracted slope and intercept were used as yields in the RSM analysis. Finally, we used the RSM model to predict the S21 and VSWR with reference to combinations of the three factors other than those mentioned above, and cross-checked the results with FEM simulations and experimental results.

Focusing on the results, the proposed RSM model fitted very accurately the values coming from linearization of FEM simulations and experimental measurements. In particular, for the S21 coefficient parameters, the disagreement between the RSM and the physical model did not exceed the 7% for the slope and the 4% for the intercept. Similar spreads were detected for the VSWR parameter slope and intercept. Concerning the validation against experimental data, the RSM was effective in predicting both the S21 slope and intercept within an error of 3.5%. This was also confirmed by the value of R2 that was close to 1, thus indicating a direct relationship between the physical and the RSM model. It has also to be reminded that the proposed method allowed us to predict the value without running a simulation but simply by substituting the factor values into the model equations.

In light of the validated effectiveness reported in this work, the RSM approach admits significant room for being extended, so that the main significant DoFs in the involved physical domains (mechanical; electrostatic; RF) can be grouped and analyzed together, leading to fast and efficient optimization of rather complex problems.

In fact, the analytical study of physical problems referred to complex geometries, is a difficult task. In several cases, extrapolating a global physical equation that describes the whole system is almost impossible. The system at stake is analytically understandable only for the single elements that compose it, and to globally resolve the physical problem, FEM tools are commonly employed. However, one of the disadvantages of FEM is the lack of a direct relationship between the outcome and the geometrical parameters. On the other hand, using RSM, after a set of physical FEM simulations, allowed us to partially recover the understanding of the dependences between the outcome and the geometry, by means of empirical equations.

## Supplementary Information

Supplementary Tables.
