# Supplementary material for: Exploitation of response surface method for the optimization of RF-MEMS reconfigurable devices in view of future beyond-5G, 6G and super-IoT applications
Source: Sci Rep. 2022 Mar 3;12:3543. doi: 10.1038/s41598-022-07643-0 (PMC8894374; doi:10.1038/s41598-022-07643-0)
Supplement: Supplementary file 1 — Supplementary Tables. [file 41598_2022_7643_MOESM1_ESM.pdf]

– Supporting Information –

Exploitation of Response Surface Method for the Optimization of RF-MEMS Reconfigurable Devices in View of Future Beyond-5G, 6G and Super-IoT Applications

Run	Factor 1 A:R_{SQ} [Ω/sq]	Factor 2 B:W_{SER} [mm]	Factor 3 C:W_{SHT} [mm]	Response 1 S21 Intercept [dB]	Response 2 S21 Slope [dB/GHz]	Response 3 VSWR Intercept [dB]	Response 4 VSWR Slope [dB/GHz]
1	40	0.3	0.015	-12.6722	0.0593507	1.18821	0.0971603
2	40	0.3	0.025	-15.3946	0.0896057	0.972075	0.124514
3	40	0.3	0.035	-16.0889	0.0980234	0.945826	0.127135
4	40	0.4	0.015	-11.9203	0.0430969	0.995125	0.105262
5	40	0.4	0.025	-14.5788	0.0724148	0.796383	0.131821
6	40	0.4	0.035	-14.7949	0.0665327	0.671734	0.144031
7	40	0.5	0.015	-11.3753	0.0315361	0.913797	0.109097
8	40	0.5	0.025	-13.8249	0.0526102	0.680498	0.138392
9	40	0.5	0.035	-14.1946	0.0568267	0.654965	0.143011
10	100	0.3	0.015	-13.5431	0.0825557	3.68474	0.0134295
11	100	0.3	0.025	-15.7317	0.117522	3.56157	0.0256887
12	100	0.3	0.035	-16.4012	0.126478	3.63421	0.0274184
13	100	0.4	0.015	-12.6009	0.0728092	3.20041	0.0230164
14	100	0.4	0.025	-14.644	0.103617	2.97086	0.0367089
15	100	0.4	0.035	-15.0885	0.108209	2.92278	0.0410092
16	100	0.5	0.015	-11.608	0.0608491	2.71228	0.0309614
17	100	0.5	0.025	-13.5501	0.0863449	2.47846	0.0462687
18	100	0.5	0.035	-14.0788	0.0932041	2.4829	0.0486544
19	160	0.3	0.015	-15.1299	0.0880637	6.05667	-0.0308281
20	160	0.3	0.025	-16.7905	0.119469	5.91374	-0.0227481
21	160	0.3	0.035	-17.575	0.133268	6.00939	-0.0219587
22	160	0.4	0.015	-13.7599	0.0705638	5.06189	-0.0174572
23	160	0.4	0.025	-15.687	0.111181	4.95919	-0.00575274
24	160	0.4	0.035	-16.1604	0.114033	4.94912	-0.00521486
25	160	0.5	0.015	-12.8092	0.0638374	4.38884	-0.00565097
26	160	0.5	0.025	-14.4574	0.0954531	4.20304	0.0064829
27	160	0.5	0.035	-15.1001	0.103844	4.21307	0.00718444

Table 1S. Dataset used to build the RSM empirical model. The data points were obtained by simulations and extrapolation of the slope and intercept in the linear zone of the S21 and VSWR curves.

S21 Slope and Intercept model

Source	Sum of Squares	df	Mean Square	F-value	p-value	
**Model**	0.0184	12	0.0015	305.08	< 0.0001	significant
A-$\mathbf{R}_{SQ}$	0.0060	1	0.0060	1204.57	< 0.0001	***
B-$\mathbf{W}_{SER}$	0.0014	1	0.0014	282.50	< 0.0001	***
C-$\mathbf{W}_{SHT}$	0.0017	1	0.0017	347.91	< 0.0001	***
AB	0.0001	1	0.0001	13.35	0.0026	**
AC	0.0001	1	0.0001	28.32	0.0001	***
BC	0.0001	1	0.0001	15.10	0.0016	**
A^2	0.0010	1	0.0010	201.32	< 0.0001	***
B^2	0.0000	1	0.0000	2.00	0.1796	
C^2	0.0005	1	0.0005	109.00	< 0.0001	***
B^2C	0.0000	1	0.0000	2.41	0.1431	
BC^2	1.260E-06	1	1.260E-06	0.2513	0.6240	
B^2C^2	0.0001	1	0.0001	10.16	0.0066	**
**Residual**	0.0001	14	5.014E-06			
**Cor Total**	0.0184	26				

Table 2S. ANOVA table of the interpolated slope model. Several statistically not significant parameters have been inserted to maintain the model hierarchy. The levels of significance were assigned as follows: $p \leq 0.1$ (.), $p \leq 0.05$ (*), $p \leq 0.01$ (**), $p \leq 0.001$ (***).

Source	Sum of Squares	df	Mean Square	F-value	p-value	
**Model**	66.07	19	3.48	1337.78	< 0.0001	significant
A-$\mathbf{R}_{SQ}$	1.05	1	1.05	404.26	< 0.0001	***
B-$\mathbf{W}_{SER}$	4.11	1	4.11	1582.70	< 0.0001	***
C-$\mathbf{W}_{SHT}$	5.71	1	5.71	2197.72	< 0.0001	***
AB	0.4673	1	0.4673	179.77	< 0.0001	***
AC	0.0562	1	0.0562	21.63	0.0023	**
BC	0.1081	1	0.1081	41.59	0.0004	***
A^2	0.2774	1	0.2774	106.72	< 0.0001	***
B^2	0.0065	1	0.0065	2.51	0.1568	
C^2	1.68	1	1.68	645.31	< 0.0001	***
ABC	0.0246	1	0.0246	9.45	0.0180	*
A^2B	0.0271	1	0.0271	10.44	0.0144	*
A^2C	0.0105	1	0.0105	4.03	0.0847	.
AB^2	0.0028	1	0.0028	1.06	0.3377	
AC^2	0.2871	1	0.2871	110.47	< 0.0001	***
B^2C	0.0167	1	0.0167	6.42	0.0390	*
BC^2	0.0002	1	0.0002	0.0612	0.8117	
A^2B^2	0.0116	1	0.0116	4.45	0.0729	.
AB^2C	0.0127	1	0.0127	4.88	0.0629	.
B^2C^2	0.0396	1	0.0396	15.22	0.0059	**
**Residual**	0.0182	7	0.0026			
**Cor Total**	66.08	26				

Table 3S. ANOVA table of the interpolated intercept model. Several statistically not significant parameters have been inserted to maintain the model hierarchy. The levels of significance were assigned as follows: p≤0.1 (.), p≤0.05 (*), p≤0.01 (**), p≤0.001 (***).

VSWR Slope and Intercept model

Source	Sum of Squares	df	Mean Square	F-value	p-value	
**Model**	0.0906	22	0.0041	14367.56	< 0.0001	significant
A-$\mathbf{R}_{SQ}$	0.0095	1	0.0095	33026.35	< 0.0001	***
B-$\mathbf{W}_{SER}$	0.0004	1	0.0004	1320.29	< 0.0001	***
C-$\mathbf{W}_{SHT}$	0.0003	1	0.0003	1117.47	< 0.0001	***
AB	0.0001	1	0.0001	205.65	0.0001	***
AC	0.0002	1	0.0002	613.92	< 0.0001	***
BC	0.0000	1	0.0000	39.19	0.0033	**
A^2	0.0009	1	0.0009	2998.26	< 0.0001	***
B^2	1.071E-07	1	1.071E-07	0.3737	0.5740	
C^2	0.0000	1	0.0000	76.16	0.0009	***
ABC	9.315E-11	1	9.315E-11	0.0003	0.9865	
A^2B	1.191E-06	1	1.191E-06	4.16	0.1111	
A^2C	0.0000	1	0.0000	134.54	0.0003	***
AB^2	1.349E-06	1	1.349E-06	4.71	0.0958	.
AC^2	8.438E-07	1	8.438E-07	2.94	0.1613	
B^2C	0.0000	1	0.0000	41.67	0.0030	**
BC^2	1.165E-06	1	1.165E-06	4.07	0.1139	
A^2B^2	2.223E-06	1	2.223E-06	7.76	0.0496	*
A^2C^2	7.239E-06	1	7.239E-06	25.26	0.0074	**
AB^2C	4.923E-06	1	4.923E-06	17.18	0.0143	*
ABC^2	7.344E-07	1	7.344E-07	2.56	0.1846	
B^2C^2	4.387E-06	1	4.387E-06	15.31	0.0174	*
AB^2C^2	8.216E-06	1	8.216E-06	28.67	0.0059	**
**Residual**	1.146E-06	4	2.865E-07			
**Cor Total**	0.0906	26				

Table 4S. ANOVA table of the interpolated slope model. Several statistically not significant parameters have been inserted to maintain the model hierarchy. The levels of significance were assigned as follows: $p \leq 0.1$ (.), $p \leq 0.05$ (*), $p \leq 0.01$ (**), $p \leq 0.001$ (***).

Source	Sum of Squares	df	Mean Square	F-value	p-value	
**Model**	86.62	18	4.81	8372.11	< 0.0001	significant
A-$\mathbf{R}_{SQ}$	26.07	1	26.07	45357.81	< 0.0001	***
B-$\mathbf{W}_{SER}$	1.03	1	1.03	1795.78	< 0.0001	***
C-$\mathbf{W}_{SHT}$	0.0488	1	0.0488	84.93	< 0.0001	***
AB	1.55	1	1.55	2703.24	< 0.0001	***
AC	0.0199	1	0.0199	34.64	0.0004	***
BC	0.0080	1	0.0080	13.91	0.0058	**
A^2	0.0316	1	0.0316	55.05	< 0.0001	***
B^2	0.0010	1	0.0010	1.80	0.2166	
C^2	0.0067	1	0.0067	11.72	0.0090	**
ABC	0.0016	1	0.0016	2.73	0.1371	
A^2B	0.0041	1	0.0041	7.05	0.0290	*
A^2C	0.0001	1	0.0001	0.0992	0.7608	
AB^2	0.0048	1	0.0048	8.34	0.0203	*
B^2C	0.0050	1	0.0050	8.66	0.0186	*
BC^2	8.599E-06	1	8.599E-06	0.0150	0.9057	
A^2B^2	0.0027	1	0.0027	4.72	0.0616	.
A^2BC	0.0019	1	0.0019	3.28	0.1076	
B^2C^2	0.0030	1	0.0030	5.23	0.0515	.
**Residual**	0.0046	8	0.0006			
**Cor Total**	86.62	26				

Table 5S. ANOVA table of the interpolated intercept model. Several statistically not significant parameters have been inserted to maintain the model hierarchy. The levels of significance were assigned as follows: $p \leq 0.1$ (.), $p \leq 0.05$ (*), $p \leq 0.01$ (**), $p \leq 0.001$ (***).
